# Doula services for Medicaid beneficiaries in Virginia: access, utilization, and policy lessons

**DOI:** 10.1093/haschl/qxaf252

**Published:** 2026-01-13

**Authors:** Desirae Leaphart Mensah, Alison E Cuellar

**Affiliations:** College of Public Health, George Mason University, Fairfax, VA 22030, United States; College of Public Health, George Mason University, Fairfax, VA 22030, United States

**Keywords:** doula, Medicaid policy, pregnant women, maternal health

## Abstract

**Objective:**

Doula support improves maternal health outcomes. States have increasingly included doula services as a covered benefit under Medicaid. We evaluated the implementation of Virginia's Medicaid doula policy and identified lessons to improve access and utilization.

**Methods:**

This mixed-methods study used administrative data (Medicaid claims data from January 2022-December 2024 and state-certified doula registry data) and qualitative interviews with 9 doula Medicaid providers to understand the barriers and facilitators to doula care among Medicaid beneficiaries in Virginia.

**Results:**

At the time of the study, there were 130 state-certified doula Medicaid providers in Virginia, but fewer than half billed Medicaid for services, most commonly billing for postpartum care. Additionally, utilization of doula services among beneficiaries was low (less than 1%) from 2022 to 2024. Geographic disparities showed limited doula availability in some high-need areas. Five policy lessons emerged.

**Conclusion:**

State Medicaid programs can strengthen doula policy implementation, and in turn strengthen the doula workforce and expand access to doula care for Medicaid beneficiaries, by revising reimbursement rates and structure to better reflect the scope of doula services, recruiting doulas in underserved areas, providing technical assistance for enrollment and billing, expanding outreach to beneficiaries, and engaging a range of doulas in policy discussions.

## Teaser

Doula care can advance maternal health for high-risk groups. As states expand Medicaid coverage for doula care, Virginia's experience offers important lessons. Drawing on Virginia's first three years of Medicaid doula coverage and the perspective of doulas themselves, this study uncovers workforce challenges, geographic disparities, and key policy recommendations to expand access and strengthen the doula model of care.

Key Takeaways

- State Medicaid agencies and managed care organizations should strengthen the workforce for doula Medicaid providers by simplifying and supporting the enrollment and reimbursement processes.
- Policymakers should prioritize recruiting doulas in areas in underserved and rural regions to promote equitable access.
- Policymakers should routinely engage doulas as partners in program design and implementation.

## Introduction

Doulas are non-medical professionals who provide peer-to-peer, perinatal support services, including evidence-based education, connections to resources, and support during delivery.^1^ The benefits of doula support are numerous for mothers and infants.^2-5^ Research also demonstrates that doula support is particularly effective for Black patients, who disproportionately face poor maternal and infant outcomes.^1^

To address rising maternal mortality, severe maternal morbidity, and racial disparities in maternal care, states are increasingly opting to include doulas as a state-licensed provider category and doula services as a benefit under their state Medicaid programs.^6^ Doula services have traditionally not been covered by private health insurance or Medicaid,^7^ reducing access for low-income women.^8^ As of January 2025, 15 states and Washington D.C. have implemented Medicaid coverage for doula services, up from 5 in 2022.^9^ Findings from the initial experiences of Oregon and Minnesota's Medicaid doula coverage identified implementation challenges related to administrative barriers for doulas,^10^ reimbursement rates,^11^ and lack of representation among doulas of the populations that need their services most.^12^

This mixed-methods study draws lessons regarding Virginia's implementation of its doula Medicaid policy. In 2022, Virginia implemented Medicaid coverage for doula support. The goals of the policy were to address maternal morbidity by expanding access to doula care during the perinatal, labor and delivery, and postpartum periods as a measure to lower costs and improve health outcomes. Additionally, Virginia emphasized the importance of community-based doulas who can provide culturally congruent care.^13^ Although most pregnant women with Medicaid coverage in Virginia are White, pregnant Black women are disproportionately more likely to be covered by Medicaid. Twenty-eight percent of pregnant Medicaid enrollees are Black compared to 20% of women of childbearing age overall. Moreover, the maternal mortality rate in Virginia for Black mothers was nearly twice as high as that of White mothers.^14^ To inform its policy, Virginia collected data on reimbursement rates nationwide and held doula workgroup meetings with doulas, providers, managed care organizations, and state agency partners, among others. Virginia also collaborated with New Jersey's state Medicaid program to glean best practices and lessons learned.^15^

To enroll as Medicaid providers in Virginia, doulas must first become state-certified through the Virginia Certification Board. They then register as network providers separately with the 5 contracted Medicaid managed care organizations (MCOs). In Virginia, 91% of pregnant Medicaid enrollees receive their benefits through an MCO.^13^ As of January 2025, Virginia's Medicaid reimbursement rate for doula support was $859 total for up to 8 prenatal/postpartum visits (up to 90 minutes for an initial prenatal visit and 60 minutes for all remaining visits) and labor support. Doulas also have 2 opportunities to earn a $50 “linkage to care” incentive payment if they complete at least one postpartum visit. The first incentive occurs if the client is seen by a clinician for a postpartum visit, and the second if the newborn is seen by a pediatrician for a newborn visit.^16^

The objective of this mixed-methods study was to derive policy lessons to inform the implementation of a doula coverage policy in Medicaid. These lessons learned can apply to Virginia as well as other states implementing or seeking to implement a similar policy.

## Data and methods

### Overview

We employed a convergent parallel design, in which both quantitative and qualitative data were collected simultaneously, analyzed independently, and then interpreted together to triangulate the findings.^17^ The study was approved by George Mason University's Institutional Review Board.

### Quantitative analysis

We used 4 quantitative data sources. The November 2024 publicly available doula enrollment data from the Virginia Certification Board Doula registry^18^ contained the number of state-certified and Medicaid-participating doulas, their race and ethnicity, and ZIP code. Publicly available data^19^ on the annual total births funded by Medicaid in each county provided important context for doula services. We further identified all Medicaid claims data for any doulas listed as state-certified or Medicaid-participating. To analyze claims data, we used doula name and address information from the registry to obtain provider identifiers from the National Plan and Provider Enumeration System NPI Registry system, and confirmed their primary classification as doulas.^20^ Claims were included from January 2022, when Medicaid doula coverage was implemented, to December 2024 to provide descriptive data on doula service volume by region, how many doulas submitted Medicaid claims for services, and what procedures doulas were providing to beneficiaries.

We used ArcGIS Pro 3.1^21^ to develop a dual-layer visualization that illustrates the number of doulas accepting Medicaid in comparison to Medicaid births. The map displays graduated symbols to indicate the number of doulas per county using data from the Virginia Certification Doula Registry. We added another layer, which includes the number of Medicaid births in each county, also using graduated symbols. Both layers place circles proportionate to the number of doulas accepting Medicaid and Medicaid births within each county/city in Virginia. We classified these data into quartiles to ensure meaningful differences between classes.

### Qualitative analysis

We conducted virtual key-informant interviews with doulas enrolled as Medicaid providers in Virginia. We randomly selected doulas from the Virginia Certification Board doula registry and emailed them. We screened for eligibility using an online form that verified age (18 years or older) and their status as a doula Medicaid provider in Virginia.

We developed the interview protocol (see Supplementary Appendix A) in consultation with a doula and informed by prior research.^7,22^ We probed on barriers and motivators for doulas to enroll as Medicaid providers, experiences with Medicaid claims processing, experiences providing services to Medicaid clients, recommendations for expanding access to doula care for Medicaid clients, and recommendations for improving the Virginia doula Medicaid program. A team member conducted interviews in January 2025. We provided doulas with a $25 electronic gift card.

The analysis of the key informant data was iterative and completed using both an inductive and deductive process of qualitative thematic analysis to assign codes to the key informant interview transcript data based on the central themes that emerged in the qualitative data. Two coders conducted the initial coding and compilation of the codebook using Dedoose version 9.2.22.^23^ Some codes were predetermined based on previous literature, and additional codes were developed while reviewing transcripts. The two coders engaged in an iterative process of coding, checking agreement, and discussing excerpts. A third researcher verified the coding process and themes to further reduce bias and establish confirmability. We continued interviews until thematic saturation, which we determined after reaching consensus that no new themes were emerging.

## Results

### Quantitative study results

There were 203 state-certified doulas in the registry, of which 130 were enrolled as Medicaid providers. Of these, 82% (*n* = 107) of doulas accepting Medicaid were Black, 6% (*n* = 8) were White, and 5% (*n* = 7) were multiracial. The remaining 7% were Asian or Pacific Islander, Native American or Alaskan Native, Hispanic or of unknown race (*n* = 8).

**Figure 1.**
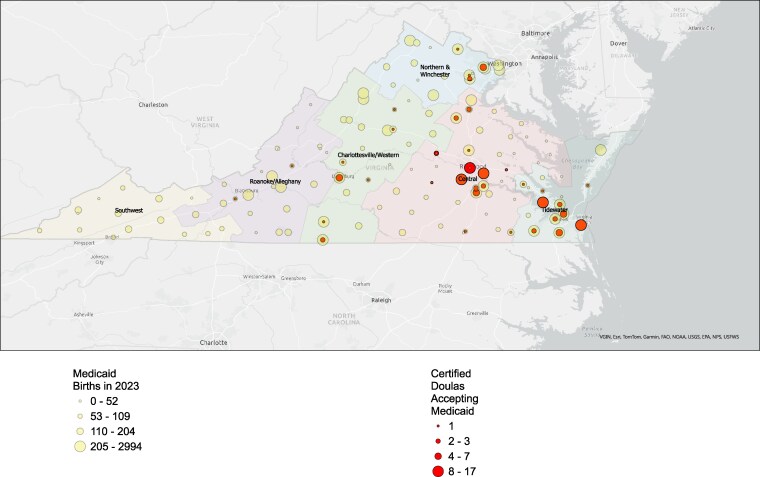
Supply and demand of doula Medicaid providers relative to where births funded by Medicaid were located [see attached supplementary file “Virginia Medicaid Doula Map.”].

The spatial analysis in Figure 1 indicated that there were several areas of high potential need (ie, higher number of Medicaid births) that overlapped with areas of higher concentrations of doulas (ie, higher number of doulas accepting Medicaid patients within an area). Notably, these included the more populated Tidewater and Central regions. There was a high need in the Northern/Winchester region of the state, with some concentrated pockets of doulas; however, the areas in this region, further away from the Washington metropolitan area, were noticeably sparser in terms of doula coverage while still having a high number of Medicaid births. There were also areas in the Charlottesville/Western and Roanoke regions of the state with high numbers of Medicaid births and little or no doula coverage. There was no coverage in the Southwest region of the state, which includes the most rural areas of Virginia; some counties and cities in this region fell within the second or third quartile of Medicaid births, indicating a coverage gap. Counties with no red circles do not have doulas who accept Medicaid (Figure 1).

Less than 1% of Medicaid pregnant women (approximately 600 total) received doula services between January 2022 and December 2024. Among the 130 Medicaid-enrolled doulas, fewer than half (*n* = 55) billed Medicaid over the first 3 years of the program. The Central region had the highest volume of Medicaid beneficiaries served (*n* = 378), the largest share of doulas submitting claims (*n* = 31; 56%), and the highest density of doulas per 1000 Medicaid births accepting Medicaid (9.9) and providing services (4.6). In contrast, other regions had much lower or no doulas accepting or providing Medicaid services, indicating a notable gap in access to doula services (Table 1).

**Table 1.** Volume of doula Medicaid participation by Virginia region, January 2022-December 2024.

Region	Medicaid beneficiaries receiving services (Jan 2022 to Dec 2024)	Doulas accepting Medicaid *N*(%)	Doulas accepting Medicaid per 1000 Medicaid births^a^	Doulas providing Medicaid Services *N*(%)	Doulas providing Medicaid services per 1000 Medicaid births^a^
Central	378	66 (51)	9.9	31 (56)	4.6
Northern/Winchester	^b^	10 (7)	0.8	^b^	^b^
Tidewater	158	38 (29)	5.9	15 (27)	2.3
Charlottesville/Western	50	14 (11)	3.7	^b^	^b^
Roanoke/Allegheny	19	2 (2)	0.8	^b^	^b^
Southwest	0	0 (0)	0	0	0
Total	∼600	130	—	55	—

^a^Based on 2023 Medicaid births.

^b^Values in Medicaid claims data that are less than 11 but greater than zero are suppressed to protect confidentiality.

We also reviewed claims data to determine which procedures were billed. Doulas most commonly billed for postpartum services in Virginia (38.5% of total procedures performed), indicating that doula services occur predominantly late in the perinatal experience. Prenatal visits represented 31.4% of services, followed by unspecified services (19.3%) and labor support (10.6%). Since labor support is a single occurrence (as opposed to multiple visits for prenatal and postpartum care), this category included a smaller proportion of services billed. Additionally, approximately one-fifth of procedures billed (19.3%) represented non-specific codes that bundled services. The heterogeneity in billing codes suggests potential challenges in the current coding structure and points to opportunities for providing clear billing guidance and more tailored reimbursement codes (Table 2). Supplementary Appendix B provides a table of the billing codes included in Table 2 and how each was categorized.

**Table 2.** Procedures billed by doulas serving Medicaid beneficiaries by region, January 2022–December 2024^a^.

	Procedure type				
Medicaid region	Prenatal visits *n* (%)	Labor support *n* (%)	Postpartum visits *n* (%)	Unspecified services *n* (%)^b^	Total procedures performed
Central	2777 (32.0)	869 (10.0)	3458 (39.9)	1566 (18.1)	8670 (100%)
Northern/Winchester	^c^	^c^	^c^	^c^	25 (100%)
Tidewater	967 (29.0)	367 (11.0)	1320 (39.6)	677 (20.3)	3331 (100%)
Charlottesville/Western	222 (29.1)	98 (12.9)	304 (39.9)	138 (18.1)	762 (100%)
Roanoke/Allegheny	179 (44.2)	65 (16.0)	0	161 (39.8)	405 (100%)
Southwest	0	0	0	0	0 (100%)
Virginia	4145 (31.4)	1399 (10.6)	5082 (38.5)	2542 (19.3)	13 193 (100%)

^a^About 5.3% of reimbursed procedures included services outside of the scope of a doula (such as psychotherapy and psychiatric evaluation), indicating processing or provider categorization errors. These procedures were excluded from Table 2.

^b^“Unspecified” most commonly includes unlisted or unspecified home visits and unlisted special services that may still be relevant to perinatal care.

^c^Values less than 11 are suppressed to protect confidentiality.

### Qualitative results

The interviewees included nine doulas, all female, based in the Central (*n* = 4), Tidewater (*n* = 3), and Northern/Winchester (*n* = 2) regions. All doulas reported providing prenatal, labor, and postpartum services. Five doulas interviewed reported being a part of a doula organization (also referred to by doulas as a “collective”).

Five themes emerged from the interviews.

### There is a high demand for doula services among Medicaid beneficiaries, with some areas of Virginia having shortages

Doulas reported that there is a high demand for doula services among Medicaid beneficiaries, but depending on their region, they had differing perspectives on the adequacy of doula availability. Doulas in the Tidewater and Northern/Winchester regions more commonly expressed that there were not enough doulas to meet the demand. For example, one doula expressed that there are no “backup” doulas available that can provide support to her Medicaid clients should she need it. Several doulas mentioned that there is a high concentration of doulas in the Richmond area (Central Region). To address the gap in access to doula care, some doulas called out the need for more awareness among doulas about the opportunity to enroll as a Medicaid provider in Virginia.

### Doulas' experiences with Medicaid enrollment varied by MCO and whether they worked independently or as part of a doula collective

Doulas reported on their experiences enrolling with the state as Medicaid providers and with the 5 Medicaid MCOs as network providers. Most doulas reported some difficulty with the enrollment process, with independent doulas tending to experience the most difficulty. Doulas who had difficulty reported a significant administrative burden with enrollment and redundancy in some of the training requirements.

Informants expressed mixed sentiments regarding the usefulness of the resources provided by the state, with some doulas suggesting that the resources should have been more tailored to doulas with limited experience with Medicaid, while others found the state website to be useful. Doulas without the support of a collective often had to navigate the complex Medicaid enrollment process independently. Doulas who were part of a collective often received support with enrollment from the collective.

It takes a lot of persistence to really get that done on your own if you're doing it independently. I feel like there's really not a lot of guidance for independent doulas to get that process completed. – (Independent Doula)

So, because I went through an agency, the process was really streamlined. – (Doula with a Collective)

Additionally, doulas reported variability in the amount of support they received from the MCOs with which they enrolled; some doulas reported enrolling with only certain MCOs due to the incremental burden of enrolling with more MCOs.

There were five different insurance providers in my area, and I ended up going through the contracting process with two of the five, and I didn't do all five just because, I mean, who has the time for that? They all had their own process. So, as a solo practice doula, that was not an option for me to do all five.

Some doulas highlighted one MCO that they felt did particularly well in supporting enrollment by providing a tailored “doula cheat sheet” and a dedicated point person for doulas.

### Medicaid awareness of covered doula services is low overall, but might be facilitated by case managers

Doulas shared their perspective that Medicaid beneficiaries may know about doulas, but not be aware of their own eligibility for doula services. Informants viewed this lack of awareness among Medicaid beneficiaries as a significant barrier to doula care. Doulas were aware that Medicaid has flyers advertising doula services to Medicaid beneficiaries, but emphasized the need for more proactive strategies. Doulas recommended more advertising of doula services through social media and greater participation by doulas in community events. They said Medicaid beneficiaries may also need support in adjusting their beliefs about doula services. Several doulas expressed that Medicaid beneficiaries may feel discouraged from seeking doula services because they might view it as a luxury they cannot afford or do not deserve.

Doulas emphasized that case managers, who use Medicaid provider databases to inform Medicaid beneficiaries about local doulas, are key facilitators of doula services. Others highlighted alternative mechanisms for raising awareness, such as word of mouth among Medicaid beneficiaries; information and referrals from MCOs; and efforts by community-based organizations to promote doula services via their websites or social media. Doulas were less likely to view healthcare providers as promoting awareness and referrals.

I think each MCO, as soon as a mom becomes pregnant, should get them connected with case management, and case management should share every single incentive with that person and let them decide which one they want to take advantage of.

Doulas also emphasized the potential of healthcare providers to facilitate doula care, both in terms of being more flexible in working with doulas and serving as advocates by educating their patients on the benefits of doula care.

More awareness and outreach, and continuing to give evidence-based information on how doulas are actually beneficial throughout the pregnancy, birth, and postpartum periods—how it's statistically proven that doulas [improve] birth outcomes. Also, holding the doctors, physicians, and hospitals accountable.

Additionally, doulas shared that Medicaid beneficiaries often begin doula services later in their pregnancies, indicating a delay in their awareness about their doula benefits.

### Doulas expressed frustration with Medicaid reimbursement limitations

Some doulas shared that submitting claims for reimbursement was more burdensome administratively than enrolling as a Medicaid provider. This was particularly the case for independent doulas rather than for those who were part of collectives that submit claims on their behalf. Doulas shared their frustrations with rejected claims and billing codes. Some doulas stated that it was often easier to move on from a denied claim rather than to waste the time and resources to fight it, resulting in no payment.

I'm always afraid I'm doing this wrong. And I'm very reluctant, like I have underpaid myself significantly, probably with what I'm even entitled to, just because I find the whole process intimidating and I'm always afraid I'm doing it wrong.

Doulas also reported that the reimbursement rate they receive from Medicaid is lower than their rate for self-paying clients and is not commensurate with their efforts. The low reimbursement rate was perceived as one of the biggest barriers to Medicaid participation. Higher rates, they felt, could lead to more doulas enrolling in Medicaid and serving more Medicaid clients. Some Medicaid-enrolled doulas supplement their incomes by having multiple jobs or ensuring they have a higher number of self-paying clients.

Most doulas reported that the Virginia Medicaid program's reimbursement structure in terms of the limits on the number and length of prenatal and postpartum visits does not align with their clients' needs; the number of visits was usually reported as too few, and the length of time was too short.

### Doulas wanted more opportunities to participate in policy discussions with Medicaid

Independent doulas reported not being involved in discussions with state Medicaid staff, but they were aware of doulas within collectives engaging in such efforts, and some believed that collectives had a stronger influence on Medicaid policy and structure. Regardless of whether they were independent or part of a collective, doulas shared a desire to be included in discussions aimed at improving Medicaid policy and to have a mechanism to convey their experiences with Virginia's Medicaid program to policymakers.

## Discussion

Based on our quantitative and qualitative findings, we offer the following policy lessons that may be useful to Virginia and other states.

### Reduce barriers to doula enrollment and participation in Medicaid

Our study revealed that a considerable number of doulas are state-certified but do not participate in Medicaid. Qualitative data suggested that higher reimbursement rates would increase participation, a recommendation consistent with the experience in other states.^7^ For example, Oregon raised its initial reimbursement rate of $350 per pregnancy to $1,500,^24^ while Minnesota increased its reimbursement rates from $411 per pregnancy in 2013 to a maximum of $3200 per pregnancy in 2024, which is among the highest in the country.^25^

In addition to payment, doulas in this study affirmed administrative barriers to enrolling as Medicaid providers.^7^ The state agency and MCOs could simplify the enrollment process or provide additional support or tailored resources to doulas and engage with collectives and doulas to understand which types of tailored support would be helpful.

### Target doula recruitment to shortage areas

More doulas are enrolled as Medicaid providers and serve Medicaid beneficiaries in the Central and Tidewater regions of Virginia; however, in the Northern/Winchester region of Virginia, there are relatively fewer doulas enrolled despite the number of Medicaid births in this region. There are also service gaps in the Charlottesville/Western, Roanoke/Allegheny, and Southwest regions. These included more rural areas of Virginia that generally have less access to maternal health services or have been classified as maternal care deserts.^26^ These findings indicate a need to recruit more doulas to enroll as Medicaid providers in the indicated service gap areas by expanding doula recruitment and exploring incentives or partnerships, perhaps with doula collectives, to encourage doulas to serve in these regions. In Virginia, there are doula-led organizations that are actively working to enroll more doulas as Medicaid providers and support their doula members in submitting Medicaid claims (eg, Urban Baby Beginnings and Birth in Color RVA). These organizations are largely located in the Central region, extending services to the Tidewater and Northern/Winchester regions.^13^ Additional investment could support the expansion of these services into more areas.

### Provide technical assistance to doulas for reimbursement

Half of the doulas who are enrolled as Medicaid providers in Virginia are not billing for services, and a substantial portion of claims from doulas who do bill Medicaid were submitted under unspecified maternal health services. The qualitative data indicated that doulas require additional support in selecting specific codes for their services in order to reduce claims denials. Doulas themselves called for additional, tailored educational resources to assist them in understanding the nuances of each MCO's processes. Such support and education could improve the accuracy of claims submission and financial participation by doulas in Medicaid. Other states that have reduced administrative and enrollment barriers include New Jersey and Massachusetts. New Jersey has designated doula guides to help doulas navigate their enrollment and claims submission processes.^27^ Massachusetts also provides a dedicated support team for doulas as well as comprehensive enrollment toolkits.^28^

### Strengthen pathways for Medicaid beneficiaries to access services

Quantitative data indicated an opportunity for more pregnant Medicaid beneficiaries to be connected to doula services, with less than 1% of beneficiaries receiving services between 2022 and 2024. This is similar to the number of beneficiaries that received doula services in Oregon between 2016 and 2020.^29^

Regarding a possible explanation, doulas stated in interviews that there were beneficiaries who were unaware of their doula benefits. Case managers, MCOs, and providers can increase the number of Medicaid beneficiaries referred to doulas; other opportunities include raising awareness through social media and community events. Doulas shared that Medicaid beneficiaries are connected to services late in their pregnancies, perhaps leading to missed opportunities for prenatal services. Medicaid beneficiaries should be educated about the benefits of doula services early in their pregnancies by their healthcare providers or case managers.

### Engage more independent and collective-based doulas in Medicaid discussions

Doulas desire to play a key role in planning and implementing efforts to expand the Virginia doula Medicaid program, both in terms of the number of doulas enrolled as Medicaid providers and the number of Medicaid beneficiaries receiving doula services. Both the quantitative and qualitative data highlight the importance of collaboration between the state Medicaid program and doulas from different regions of Virginia, including both doulas part of collectives and working independently. This is consistent with research in other states, indicating the importance of collaboration with doulas to improve policy.^11^ In the policy development process, Virginia held workgroup meetings that included community-led doula agencies and doulas of color;^13^ however, from the perspectives of doulas included in this research, more engagement with doulas that do not work with a community-led organization is needed.

### Limitations

This study's findings are preliminary and do not indicate long-term trends. Regarding the qualitative interviews, although doulas were randomly recruited, not all regions were represented in the final sample. Furthermore, while some of the findings can be applied to other states, others are specific to Virginia, given its demographic and geographic contexts and program maturity.

## Conclusion

As additional states implement Medicaid coverage for doula support, doulas' experiences enrolling as Medicaid providers and the uptake of doula services among Medicaid beneficiaries can inform policies aimed at incentivizing doula care. State Medicaid programs can strengthen doula policy implementation by recruiting doulas in underserved areas, providing more support to doulas regarding enrollment and billing processes, increasing outreach to beneficiaries, and revising reimbursement rates and structure to better reflect the scope of doula care.

## Supplementary Material

qxaf252_Supplementary_Data
