# Supplementary material for: Doula services for Medicaid beneficiaries in Virginia: access, utilization, and policy lessons
Source: Health Aff Sch. 2026 Jan 13;4(1):qxaf252. doi: 10.1093/haschl/qxaf252 (PMC12798800; doi:10.1093/haschl/qxaf252)
Supplement: qxaf252_Supplementary_Data [file qxaf252_supplementary_data.zip › Appendix B. Procedure Codes Included.docx]

**Appendix B. Procedure Codes Included in Medicaid Claims Analysis**

| Procedure  Code | Description | Category |
| --- | --- | --- |
| H1002 | Prenatal care, at-risk enhanced service; care coordination. This code is used for enhanced prenatal care services involving care coordination for at-risk patients. | Prenatal |
| S9409 | Home management of preeclampsia, includes administrative services, care coordination, and all necessary supplies and equipment; per diem. | Prenatal |
| S9425 | Home infusion therapy, pain management infusion; per diem. | Unspecified |
| Z32.2 | Encounter for childbirth instruction. | Prenatal |
| 0500F | Initial prenatal care visit. | Prenatal |
| 0503F | Postpartum care visit. | Postpartum |
| 59400 | Routine obstetric care including antepartum care, vaginal delivery (with or without episiotomy, and/or forceps) and postpartum care | Unspecified |
| 59409 | Vaginal delivery only (with or without episiotomy and/or forceps). | Delivery |
| 59414 | Delivery of placenta (separate procedure). This code is used for the delivery of the placenta when performed separately from the delivery of the baby. | Delivery |
| 59425 | Antepartum care only; 4-6 visits. This code is used when a provider delivers only antepartum care consisting of 4 to 6 visits, without performing delivery or postpartum care. | Prenatal |
| 59430 | Postpartum care only (separate procedure). This code is used for postpartum care services provided separately from delivery. | Postpartum |
| 59514 | Cesarean delivery only. This code is used for a cesarean section delivery without postpartum care. | Delivery |
| 59515 | Cesarean delivery only; including postpartum care. This code is used for a cesarean section delivery with postpartum care. | Delivery |
| 59620 | Cesarean delivery only, following attempted vaginal delivery after previous cesarean delivery. | Delivery |
| 97199 | Unlisted physical medicine/rehabilitation service or procedure. This code is used for physical medicine or rehabilitation services not otherwise specified. | Unspecified |
| 99199 | Unlisted special service, procedure or report. This code is used for services or procedures that do not have a specific code. | Unspecified |
| 99425 | Initial neonatal intensive care, per day, for the evaluation and management of a critically ill neonate or infant. This code is used for the initial day of intensive care for a critically ill neonate or infant. | Postpartum |
| 99500 | Home visit for prenatal monitoring and assessment, including fetal heart rate monitoring. | Prenatal |
| 99600 | Unlisted home visit service or procedure. This code is used for home visit services or procedures that are not otherwise specified. | Unspecified |
