# Supplementary material for: Doula services for Medicaid beneficiaries in Virginia: access, utilization, and policy lessons
Source: Health Aff Sch. 2026 Jan 13;4(1):qxaf252. doi: 10.1093/haschl/qxaf252 (PMC12798800; doi:10.1093/haschl/qxaf252)
Supplement: qxaf252_Supplementary_Data [file qxaf252_supplementary_data.zip › Appendix Key Informant Interview Guide_10.7.25.docx]

**Appendix. Key Informant Interview Guide**

Introduction

First, we'll talk about your background as a doula and your general experiences in this role.

1. How long have you been a doula?
2. Where do you practice?
3. What types of services do you provide?
4. What inspired you to become a doula, and what motivates you in this work?

Enrolling as a Medicaid Provider

Next, we'll focus on your experience enrolling as a Medicaid provider, including any challenges or benefits you've encountered.

1. Why did you decide to become a Medicaid Provider?
2. What are the challenges with enrolling to become a Medicaid provider in Virginia?
3. Can you share a specific moment or experience that illustrates these challenges?
4. Did you receive any support or guidance during the enrollment process? What kind of support would have helped?
5. What are some things you enjoy about providing services to Medicaid patients?

Serving Medicaid Beneficiaries

Finally, we'll discuss your work with Medicaid clients, including how you connect with them and the support you receive from the state in serving them.

1. How are people with Medicaid referred to you?
2. Have you encountered situations where it wasn't clear if a client was a Medicaid beneficiary? How do you navigate this?
3. How do your Medicaid clients become aware of your services?
4. Is there a high demand for doula care among Medicaid beneficiaries? Are there enough doulas to meet the need?
5. Have you experienced any differences in serving Medicaid patients in comparison to your clients who are not Medicaid beneficiaries? How are they different? How are they the same?
6. Have you observed differences in outcomes or satisfaction among your Medicaid clients compared to others?
7. What has been your experience as a doula provider that serves Medicaid patients in terms of administrative work (i.e., paperwork)? Are there specific aspects of the paperwork that are especially challenging? Why?
8. How does the state support you with the administrative hurdles?
9. What's it like working with other hospital staff and providers to support Medicaid beneficiaries?
10. Are state-approved doula training and certification organizations tailored to train doulas to meet the needs of Medicaid beneficiaries? What trainings are needed?

Policy and Systems

1. Do doulas have a say in developing the policy to provide Medicaid coverage for doula support in the state? What has been your experience?
2. What challenges in the Medicaid system or healthcare system make it harder to serve Medicaid clients?

Closing

1. What would need to happen to ensure more doulas are able to enroll as Medicaid providers?
2. What would need to happen to ensure that more Medicaid beneficiaries are connected to doula services?
3. Is there anything else you would like to share regarding your experience as a doula about becoming a Medicaid provider or serving Medicaid patients?
